# Study of Oxidative Stress in Human Lens Epithelial Cells Exposed to 1.8 GHz Radiofrequency Fields

**DOI:** 10.1371/journal.pone.0072370

**Published:** 2013-08-26

**Authors:** Shuang Ni, Yibo Yu, Yidong Zhang, Wei Wu, Kairan Lai, Ke Yao

**Affiliations:** 1 Eye Center, Second Affiliated Second Hospital of Zhejiang University School of medicine, Hangzhou, Zhejiang, China; 2 Zhejiang Provincial Key Lab of Ophthalmology, Hangzhou, Zhejiang, China; St. Georges University of London, United Kingdom

## Abstract

**Objectives:**

The aims of the present study were to determine oxidative stress and to explore possible reasons of reactive oxygen species (ROS) increase in human lens epithelial (HLE) B3 cells exposed to low intensity 1.8 GHz radiofrequency fields (RF).

**Methods:**

The HLE B3 cells were divided into RF exposure and RF sham-exposure groups. The RF exposure intensity was at specific absorption rate (SAR) of 2, 3, or 4 W/kg. The ROS levels were measured by a fluorescent probe 2′7′-dichlorofluorescin diacetate (DCFH-DA) assay in the HLE B3 cells exposed to 1.8 GHz RF for 0.5, 1, and 1.5 h. Lipid peroxidation and cellular viability were detected by an MDA test and Cell Counting Kit-8 (CCK-8) assays, respectively, in the HLE B3 cells exposed to 1.8 GHz RF for 6, 12, and 24 h, respectively. The mRNA expression of *SOD1*, *SOD2*, *CAT,* and *GPx1* genes and the expression of SOD1, SOD2, CAT, and GPx1 proteins was measured by qRT-PCR and Western blot assays in the HLE B3 cells exposed to 1.8 GHz RF for 1 h.

**Results:**

The ROS and MDA levels significantly increased (*P*<0.05) in the RF exposure group and that the cellular viability, mRNA expression of four genes, and expression of four proteins significantly decreased (*P*<0.05) compared with the RF sham-exposure group.

**Conclusions:**

Oxidative stress is present in HLE B3 cells exposed to 1.8 GHz low-intensity RF and that the increased production of ROS may be related to down-regulation of four antioxidant enzyme genes induced by RF exposure.

## Introduction

According to statistics released by the International Telecommunication Union in June 2012, total cellular phone subscriptions have reached almost 6 billion by end 2011, corresponding to global penetration of 86%. A cellular phone network consists of two communicating elements: cellular phones and base stations. Their frequencies vary according to the specific system but are nowadays usually around 900 or 1 800 MHz (GSM) and 2 200 MHz (UMTS), which are in the microwave range. In the general population, concern about possible adverse health effects induced by radiofrequency fields (RF) is fast growing owing to increasing exposure to radiation from cellular phones and base stations, together with exposure to other sources of nonionizing radiation such as power lines and radar. For example, in cytogenetic biomonitoring studies of RF-exposed humans, the majority of studies showed that RF-exposed individuals have increased frequencies of genetic damage (e.g., chromosomal aberrations) in their lymphocytes or exfoliated buccal cells [1]. Some studies also detected an association between human health and exposure to RF, with clinical conditions including childhood leukaemia, brain tumors, genotoxicity, and neurodegenerative disease reported [1], [2].

It is well known that oxidative stress, defined as an imbalance between oxidants and antioxidants in favor of the former, results in many biochemical changes and also is an important factor contributing to several human chronic conditions such as atherosclerosis, cardiovascular diseases, neurodegenerative disorders, and cancer; it is also associated with the aging process [3]. Many studies of oxidative stress associated with RF exposure in vivo or in vitro have been conducted [1]. However, these have yielded inconsistent results, with several in vivo and in vitro investigations indicating that RF exposure could increase ROS levels [2]–[5], but others reporting that RF exposure did not increase spontaneous ROS formation [6]–[9]. Oxidative stress has been implicated in many ophthalmological disorders, e.g., senile cataracts, age-related macular degeneration of the retina, and dry eye disease [10]–[12]. As the eyeball is an important organ frequently exposed to RF from cellular phones, and biological effects of low-power microwave radiation on ocular lens and lens epithelial cells (LECs) have been reported [13], it is necessary to determine whether exposure to low-intensity RF can result in oxidative stress in ocular cells, especially LECs.

The consequences of oxidative stress can be measured by markers of damage. Malondialdehyde (MDA) is a member of a family of final products of lipid peroxidation, and is one of the most studied markers of oxidative stress [4], [14]–[21]. Reactive oxygen species (ROS) act as subcellular messengers in such complex processes as mitogenic signal transduction, gene expression, and regulation of cell proliferation^20^. The cellular proliferation and viability of LECs can be detected by a Cell Counting Kit-8 (CCK-8) assay [21], [22].

The mechanism of RF induction of ROS is not clear. Oxidative stress is due to a disruption of the balance between oxidants and antioxidants. The antioxidant defense system includes enzymes such as superoxide dismutase (SOD), glutathione peroxidase (GPx), and catalase (CAT). SOD converts O_2_^•–^ into the reactive oxygen intermediate H_2_O_2_, CAT detoxifies H_2_O_2_, and GPx catalyses the breakdown of H_2_O_2_ and lipid hydroperoxides into nontoxic products [4], [19]. It was supposed that RF exposure might affect the expression of antioxidant genes (*SOD1, SOD2*, *CAT,* and *GPX1* genes) if ROS is enhanced in human LECs exposed to RF. To shed light on this issue, it is necessary to explore possible causes of RF induced ROS at the molecular biological level.

The aims of the present study were to determine oxidative stress using 2′7′-dichlorofluorescin diacetate (DCFH-DA), MDA, and CCK-8 assays, to measure the expression levels of *SOD1*, *SOD2*, *CAT,* and *GPX1* genes by qRT-PCR and Western blot assays, and to explore possible reasons for the increase in ROS in human LECs exposed to low-intensity RF according to the guidelines of the International Commission on Non-Ionizing Radiation Protection [23].

## Materials and Methods

### Cell Culture and Exposure

Human lens epithelial (HLE B3) cells provided by professor Marjorie F. Lou [24] (University of Nebraska-Lincoln, Nebraska, U.S.A.), which were firstly established and used as a model system for investigating human lens epithelial physiology and cataract by Andley et al. [25]. HLE B3 cells were cultured in minimum essential media supplemented with 20% fetal bovine serum and 50 µg/ml gentamicin in a humidified 5% CO_2_ atmosphere at 37°C. This immortalized cell line (HLE B3) was derived from human lens infant tissue and transformed with adenovirus 12-simian virus (SV40). HLE B3 was sensitive to RF [26] and usually used for studying oxidative stress [27], [28]. The cells were divided into RF exposure groups and sham-exposure groups at the SAR of 2, 3, and 4 W/kg.

### RF Exposure System

The exposure system “sXc1800” designed by the Foundation for Information Technologies in Society (IT’IS Foundation, Zurich, Switzerland) was described by Xu et al. [29]. It consists of an exposure-waveguide chamber, a sham-exposure chamber, a signal tower, amplifiers, PC with software, and a suitable CO_2_ incubator. The whole exposure was controlled by software. The RF simulating the GSM 1.8 GHz signal was amplitude-modulated by a rectangular pulse with a repetition frequency of 217 Hz and a duty cycle of 1∶8. The cells were intermittently (5 min fields on/10 min fields off) exposed or sham-exposed to RF for different times at an average SAR of 2, 3, or 4 W/kg. The temperature rise in cells exposed to RF was less than 0.025°C/(W/kg) and therefore the temperature difference between shame-exposure group and exposure group never exceeded 0.1°C in any of the experiments.

### Intracellular ROS Detection

The intracellular ROS was quantified basically via the method described by Braicu et al. [30]. The 2′7′-dichlorofluorescin diacetate (DCFH-DA), a fluorescent probe, diffuses quickly through the cell membrane, and it is enzymatically hydrolyzed by intracellular esterases to nonfluorescent dichlorofluorescin (DCFH), which is rapidly oxidized to highly fluorescent DCF in the presence of intracellular ROS. The HLE B3 cells were seeded in 35 mm dishes (NUNC, Roskilde, Denmark, 2×10^5^ cells/dish, 2 ml culture medium) for 24 h and then exposed or sham-exposed to RF for 0.5 h, 1 h, or 1.5 h at an average SAR of 2, 3, or 4 W/kg, respectively. After exposure, the cells were washed twice with phosphate buffered saline (PBS) and incubated with culture medium containing 20 µM DCFH-DA (Sigma, Sigma-Aldrich Co. LLC.,St Louis, MO, U.S.A) for 30 min at 37°C in the dark. The cells were then washed twice by PBS and collected with trypsin-EDTA solution. After centrifugation at 1 500 rpm for 5 min, the supernatant was discarded, and the pellet was suspended in 200 µl PBS. The fluorescence intensity was measured by a multimode microplate reader (Infinite M200, Tecan, Switzerland), with excitation at 485 nm and emission at 538 nm. The fluorescence intensity ratio between the exposed cells and the sham-exposed cells served as the index.

### Cellular Viability Assay

The cellular viability was evaluated using a CCK-8 kit (Dojindo, Dojindo Molecular Technologies, Inc. Kumamoto, Japan) as described by Zhou et al. [31]. The HLE B3 cells were seeded in 35 mm dishes (1×10^5^ cells/dish, 2 ml culture medium) for 24 h, then exposed or sham-exposed to RF for 6 h, 12 h, or 24 h at an average SAR of 2, 3, or 4 W/kg, respectively. Another three dishes of cells were simultaneously incubated in a CO_2_ incubator for 6 h, 12 h, or 24 h as normal controls and used to calculate the cellular viability. After the radiation exposure, the cells were washed twice with PBS and incubated with 1 ml culture medium, which contained 10% CCK-8 solution, for 1 h at 37°C. Then, 100 µl of the culture medium from each dish was transferred into each well of a 96-well plate. The absorbance was measured by a multimode microplate reader (Infinite M200, Tecan, Switzerland) at 450 nm. The cellular viability (%) was calculated using the formula: [(As −Ab)/(Ac −Ab)]×100%. As: the absorbance of the well containing supernatant from exposure or sham-exposure dishes; Ac: the absorbance of the well containing supernatant from the normal control; Ab: the absorbance of the well containing culture medium with 10% CCK-8 solution.

### Lipid Peroxidation Assay

The extracellular MDA levels were measured via the thiobarbituric acid reactive substance test, a technique commonly used in lipid peroxidation studies [32]. The HLE B3 cells were seeded in 35 mm dishes (1×10^5^ cells/dish, 2 ml culture medium) for 24 h and then exposed or sham-exposed to RF for 6 h, 12 h, or 24 h at an average SAR of 2, 3, or 4 W/kg, respectively. MDA levels in the cell culture medium were evaluated using reagent kits (Jiancheng, Jiancheng Bioengineering Co. Ltd., Nanjing, China) according to the manufacturer’s protocol. The absorbance was determined at a wavelength of 532 nm with a multimode microplate reader (Infinite M200, Tecan, Switzerland).

### qRT-PCR Assay

The HLE B3 cells were seeded in 35 mm dishes (2×10^5^ cells/dish, 2 ml culture medium) for 24 h and then exposed or sham-exposed to RF for 1 h at an average SAR of 2, 3, or 4 W/kg, respectively. Total RNA from the HLE B3 cells was extracted using Trizol reagent (Invitrogen, Life Technologies Corporation, Carlsbad, CA, U.S.A.) according to the manufacturer’s protocol. The RNA was quantified with a spectrophotometer (Nano Drop 2000c, Thermo, U.S.A.) and stored at −80°C. Then, 1 µg of isolated RNA was revere-transcribed to cDNA using a PrimeScript® RT reagent Kit with gDNA Eraser kits (Takara, Takara Biotechnology Dalian Co. Ltd., Dalian, China) according to the manufacturer’s specification. The qRT-PCR assay was performed in an ABI 7900 HT system in a 20 µl sized reaction containing 10 µl of SYBR® Premix Ex Taq™ (Takara, Takara Biotechnology Dalian Co. Ltd., Dalian, China), 0.5 µM of PCR forward primer and reserve prime, 0.4 µl of ROX Reference Dye(50X) (Takara, Takara Biotechnology Dalian Co. Ltd., Dalian, China), and 2 µl of cDNA template. Four genes were selected for the analysis: *SOD1*, *SOD2*, *CAT,* and *GPX1.* The housekeeping gene glyceraldehyde-3-phosphate dehydrogenase (*GAPDH)* was used for normalization. Primers for *SOD1* (5′-AGGCCCCTTAACTCATCT-3′ and 5′-CTACAGGTACTTTAAAGCAACTCT-3′), *SOD2* (5′-GCACTAGCAGCATGTTGAGC-3′ and 5′-GCGTTGATGTGAGGTTCCAG-3′), *CAT* (5′-TTTCCCAGGAAGATCCTGAC-3′ and 5′-ACCTTGGTGAGATCGAATGG-3′), *GPX1* (5′-CAGTCGGTGTATGCCTTCTCG-3′ and 5′-GAGGGACGCCACATTCTCG-3′), and *GAPDH* (5′-TGCACCACCAACTGCTTAGC-3′ and 5′-GGCATGGACTGTGGTCATGAG-3′) were synthesized at Sangon Biotech (Shanghai, China). The method reported by Livak [33] was used to ensure that the amplification efficiency of each target gene was similar to that of GAPDH. The thermo-cycler program of RT-PCR was denaturation at 95°C for 30 s, followed by 40 cycles of denaturation at 95°C for 5 s and extension at 60°C for 30 s. After amplification, a melt curve was conducted to confirm that multiple specific products were amplified in these reactions. A Ct value (threshold cycle marking the cycle when the fluorescence of a given sample significantly exceeded the baseline signal) was used to calculate the fold-regulation by subtracting the Ct value for GAPDH from the Ct value for the target genes and comparing the exposure (E) results with the sham-exposure (SE) results using the following equation: fold-upregulation = 2^−ΔΔCt^, where ΔΔCt = ΔE–ΔSE, ΔE = Ct_E target_–Ct_E GAPDH,_ ΔSE = Ct_SE target_–Ct_SE GAPDH_ [33].

### Western Blot Assay

The HLE B3 cells were seeded in 35 mm dishes (2×10^5^ cells/dish, 2 ml culture medium) for 24 h and then exposed or sham-exposed to RF for 1 h at an average SAR of 2, 3, or 4 W/kg, respectively. After radiation, the cells were washed twice with ice-cold PBS. Then total protein of the HLE B3 cells was extracted with lysis buffer containing 50 mM Tris (pH 7.4), 150 mM NaCl, 1% Triton X-100, 1% sodium deoxycholate, 0.1% sodium dodechyl sulfate (SDS), sodium orthovanadate, sodium fluoride, EDTA, aprotinin, pepstantin, and leupeptin. After incubating on ice for 30 min, the extracts were centrifuged at 16 000*×g* at 4°C for 10 min, and the supernatant of each sample was transferred to a fresh tube and stored at −80°C. The protein concentration was measured by BCA assay kits (Applygen, Applygen Technologies Inc. Beijing, China). Fifteen micrograms of protein from each sample were boiled with a protein-loading buffer (Sangon Biotech, Sangon Biotech Co., Ltd., Shanghai, China) and separated on 15% SDS-polyacrylamide gels. They were then transferred to polyvinylidene difluoride membranes (Millipore, Millipore Corporation, Billerica, MA, U.S.A.). The membranes were blocked by Tris-buffered saline with 5% nonfat dry milk and then incubated with various primary antibodies (1∶1 000) for 2 h at room temperature. The primary antibodies were as follows: anti-SOD1 rabbit monoclonal antibody (Epitomics, Epitomics, Inc., California, U.S.A.), anti- SOD2 rabbit monoclonal antibody (Epitomics, Epitomics, Inc., California, U.S.A.), anti-CAT rabbit monoclonal antibody (Epitomics, Epitomics, Inc., California, U.S.A.), anti-GPX1 rabbit monoclonal antibody (Epitomics, Epitomics, Inc., California, U.S.A.), and anti-GAPDH mouse polyclonal antibody (Santa cruz, Santa Cruz Biotechnology, Inc., U.S.A). The membranes were washed for 5 min in Tris-buffered saline with 0.1% Tween-20 (TBST) for 3 times then incubated with fluorescent secondary antibodies (1∶10 000, LI-COR Biosciences, Nebraska, U.S.A.) at room temperature for 1 h. After washing twice in TBST and once in PBS, blots were detected and analyzed by the Odyssey ® Imaging system (LI-COR Biosciences, Nebraska, U.S.A.). The target protein expression levels were normalized with respect to GAPDH expression.

### Statistical Analysis

Each experiment was repeated at least three times. The data were expressed as mean ± SD. The differences in the data between the exposure groups and the corresponding sham-exposure groups were statistically analyzed with the *t* test using the SPSS statistical software version 20.0 (SPSS Inc, Chicago, U.S.A.). A *P* value less than 0.05 was considered a statistically significant difference.

## Results

### Intracellular ROS Detection

**Figure 1:**
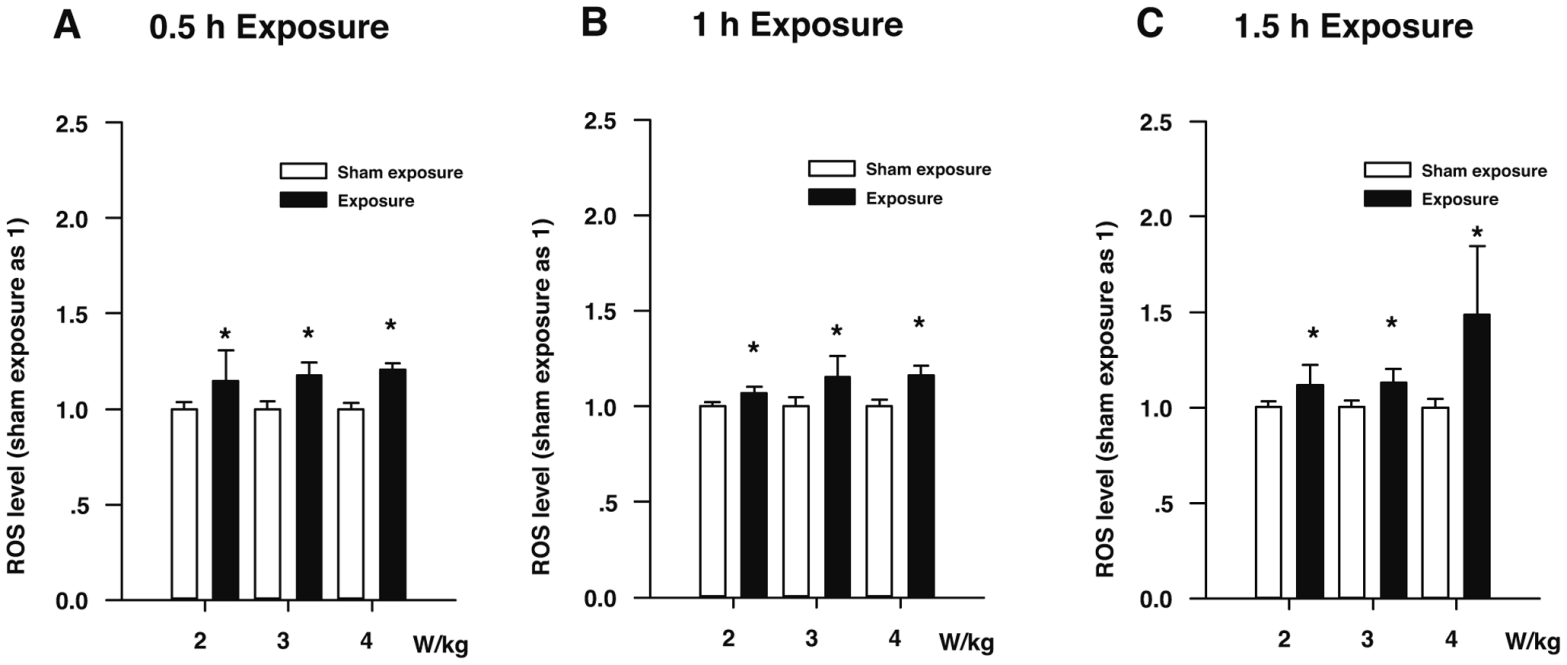
The ROS levels of the HLE B3 cells exposed to RF. (**A**) The ROS levels of the HLE B3 cells exposed to RF (SAR: 2, 3, or 4 W/kg) for 0.5 h. (**B**) The ROS levels of the HLE B3 cells exposed to RF (SAR: 2, 3, or 4 W/kg) for 1 h. (**C**) The ROS levels of the HLE B3 cells exposed to RF (SAR: 2, 3, or 4 W/kg) for 1.5 h. *P<0.05, as compared with the corresponding sham-exposure subgroups.

Figure 1 shows the ROS levels of the HLE B3 cells exposed to RF. Figure 1A indicates that the ROS levels of the HLE B3 cells exposed to RF (SAR: 2, 3, and 4 W/kg) for 0.5 h significantly increased compared with the corresponding sham-exposure HLE B3 cells (*P*<0.05). Figure 1B shows that the ROS levels of the HLE B3 cells exposed to RF (SAR: 2, 3, and 4 W/kg) for 1 h were higher than those of the corresponding sham-exposure HLE B3 cells (*P*<0.05). Figure 1C demonstrates that when compared with the corresponding sham-exposure HLE B3 cells, the ROS levels of the HLE B3 cells exposed to RF (SAR: 2, 3, and 4 W/kg) for 1.5 h were increased, especially in the subgroup exposed to 4 W/kg RF (*P*<0.05).

### Cellular Viability Assay

**Figure 2:**
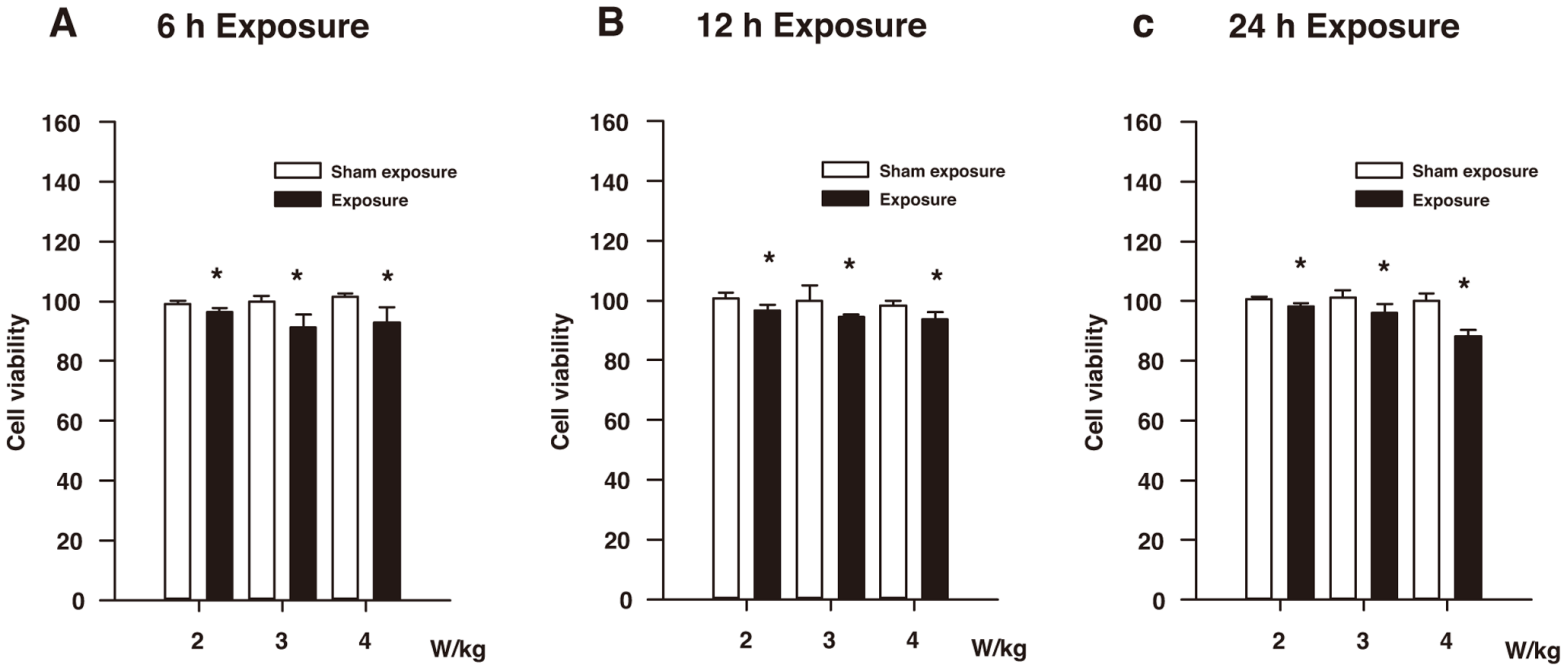
Results of CCK-8 assay of the HLE B3 cells exposed to RF. (**A**) The cellular viability of the HLE B3 cells exposed to RF (SAR: 2, 3, or 4 W/kg) for 6 h. (**B**) The cellular viability of the HLE B3 cells exposed to RF (SAR: 2, 3, or 4 W/kg) for 12 h. (**C**) The cellular viability of the HLE B3 cells exposed to RF (SAR: 2, 3, or 4 W/kg) for 24 h. *P<0.05, as compared with the corresponding sham-exposure subgroups.

The results of the CCK-8 assay are shown in Figure 2. In Figure 2A, the cellular viability of the two subgroups exposed to RF (SAR: 3 and 4 W/kg) for 6 h was significantly lower than that of the two corresponding sham-exposure subgroups (*P*<0.05). Although the difference in the cellular viability between the 2 W/kg exposure subgroup and the corresponding sham-exposure subgroup did not seem to vary greatly, the difference was still statistically significant (*P*<0.05). As shown in Figure 2B, the cellular viability of three subgroups exposed to RF (SAR: 2, 3, and 4 W/kg) for 12 h is obviously diminished compared with the three corresponding sham-exposure subgroups (*P*<0.05). In Figure 2C, the cellular viability of the two subgroups exposed to RF (SAR: 3 and 4 W/kg) for 24 h is significantly decreased compared with the two corresponding sham-exposure subgroups (*P*<0.05). In the 2 W/kg exposure subgroup for 24 h, the difference in the cellular viability between the exposure subgroup and the corresponding sham-exposure subgroup did not seem to be particularly high, but the difference was statistically significant (*P*<0.05).

### Lipid Peroxidation Assay

**Figure 3:**
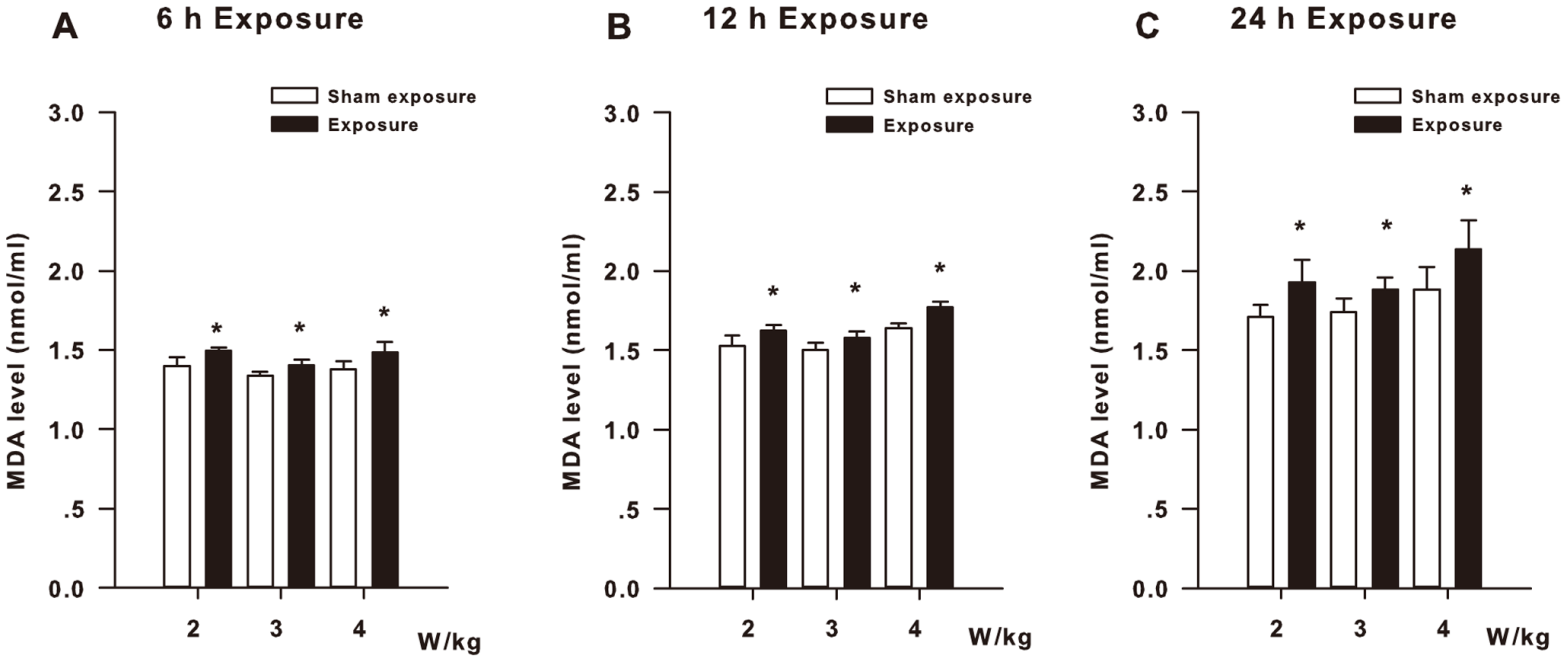
MDA levels of the HLE B3 cells exposed to RF. (**A**) The MDA levels of the HLE B3 cells exposed to RF (SAR: 2, 3, or 4 W/kg) for 6 h. (**B**) The MDA levels of the HLE B3 cells exposed to RF (SAR: 2, 3, or 4 W/kg) for 12 h. (**C**) The MDA levels of the HLE B3 cells exposed to RF (SAR: 2, 3, or 4 W/kg) for 24 h. *P<0.05, as compared with the corresponding sham-exposure subgroups.

Figure 3 shows the extracellular MDA levels of the HLE B3 cells exposed to RF. When the HLE B3 cells were exposed to RF for 6 h, the MDA levels of the three exposure subgroups (SAR: 2, 3, and 4 W/kg) were significantly higher than those of the three corresponding sham-exposure subgroups (*P*<0.05, Figure 3A). The HLE B3 cells exposed to RF for 12 h showed significant differences in the MDA levels between the three exposure subgroups (SAR: 2, 3, and 4 W/kg) and the three corresponding sham-exposure subgroups (*P*<0.05, Figure 3B). In addition, as shown in Figure 3C, when the HLE B3 cells were exposed to RF for 24 h, the MDA levels of the three exposure subgroups (SAR: 2, 3, and 4 W/kg) are obviously elevated compared with the three corresponding sham-exposure subgroups (*P*<0.05).

### mRNA Expression of Four Antioxidant Enzyme Genes

**Figure 4:**
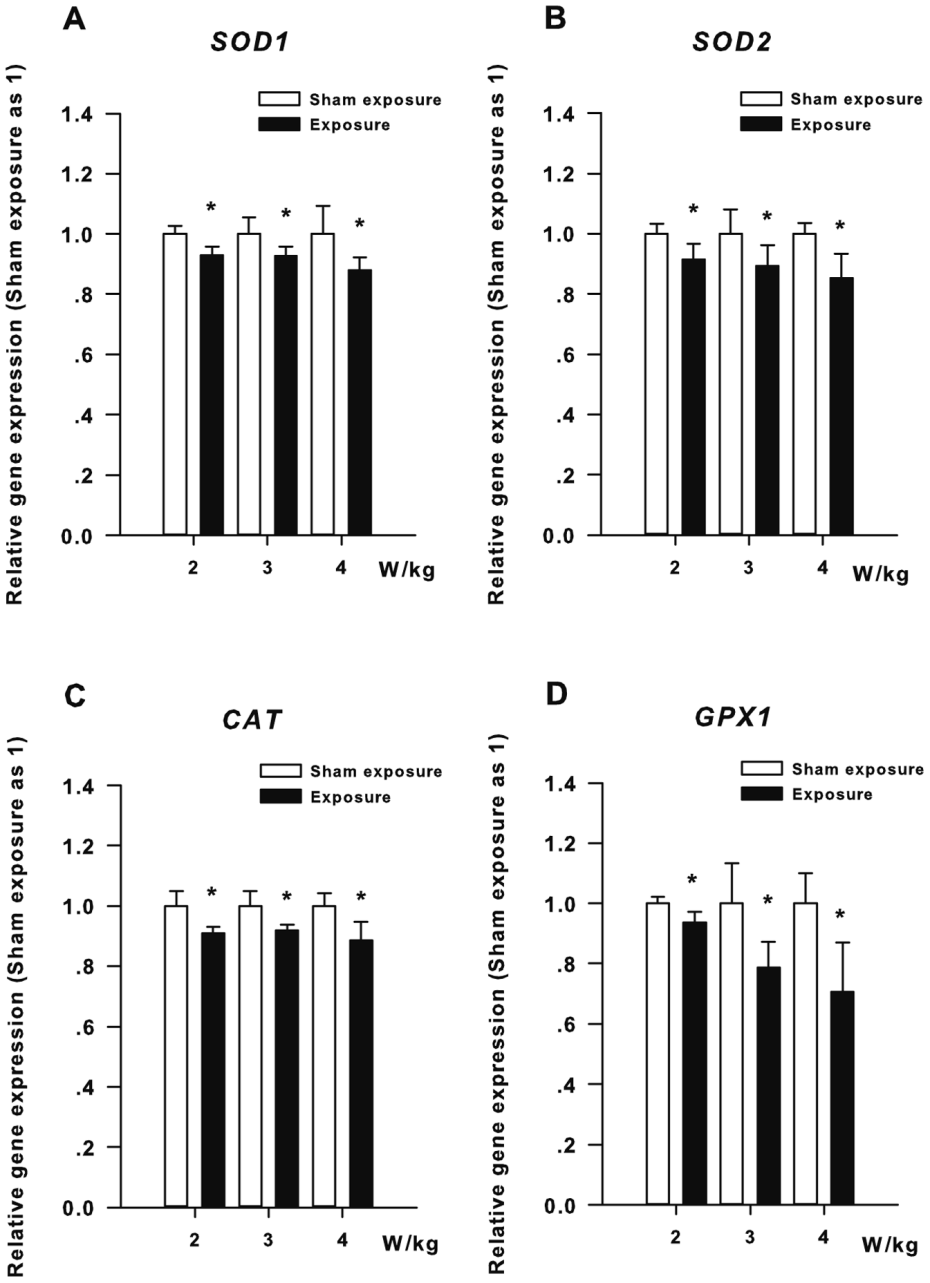
Results of qRT-PCR assay of the HLE B3 cells exposed to RF. (**A**) The expression levels of *SOD1* mRNA in the HLE B3 cells exposed to RF (SAR: 2, 3, or 4 W/kg) for 1 h. (**B**) The expression levels of *SOD2* mRNA in the HLE B3 cells exposed to RF (SAR: 2, 3, or 4 W/kg) for 1 h. (**C**) The expression levels of *CAT* mRNA in the HLE B3 cells exposed to RF (SAR: 2, 3, or 4 W/kg) for 1 h. (**D**) The expression levels of *GPX1* mRNA in the HLE B3 cells exposed to RF (SAR: 2, 3, or 4 W/kg) for 1 h. *P<0.05, as compared with the corresponding sham-exposure subgroups.

Figure 4 shows the relative mRNA expression levels of four antioxidant enzyme genes in the HLE B3 cells exposed to RF for 1 h. As apparent in Figure 4A, the *SOD1* gene expression levels of the three subgroups exposed to RF (SAR: 2, 3, and 4 W/kg) are significantly decreased compared with the three corresponding sham-exposure subgroups (*P*<0.05). Figure 4B shows that the *SOD2* gene expression levels of the three subgroups exposed to RF (SAR: 2, 3, and 4 W/kg) are significantly lower than those of the three corresponding sham-exposure subgroups (*P*<0.05). In Figure 4C, the *CAT* gene expression levels of the three subgroups exposed to RF (SAR: 2, 3, and 4 W/kg) are obviously reduced compared with the three corresponding sham-exposure subgroups (*P*<0.05). Figure 4D also reveals significant differences in the expression levels of the *GPX1* gene between the three subgroups exposed to RF (SAR: 2, 3, and 4 W/kg) and the three corresponding sham-exposure subgroups (*P*<0.05).

### Western Blot Assay

**Figure 5:**
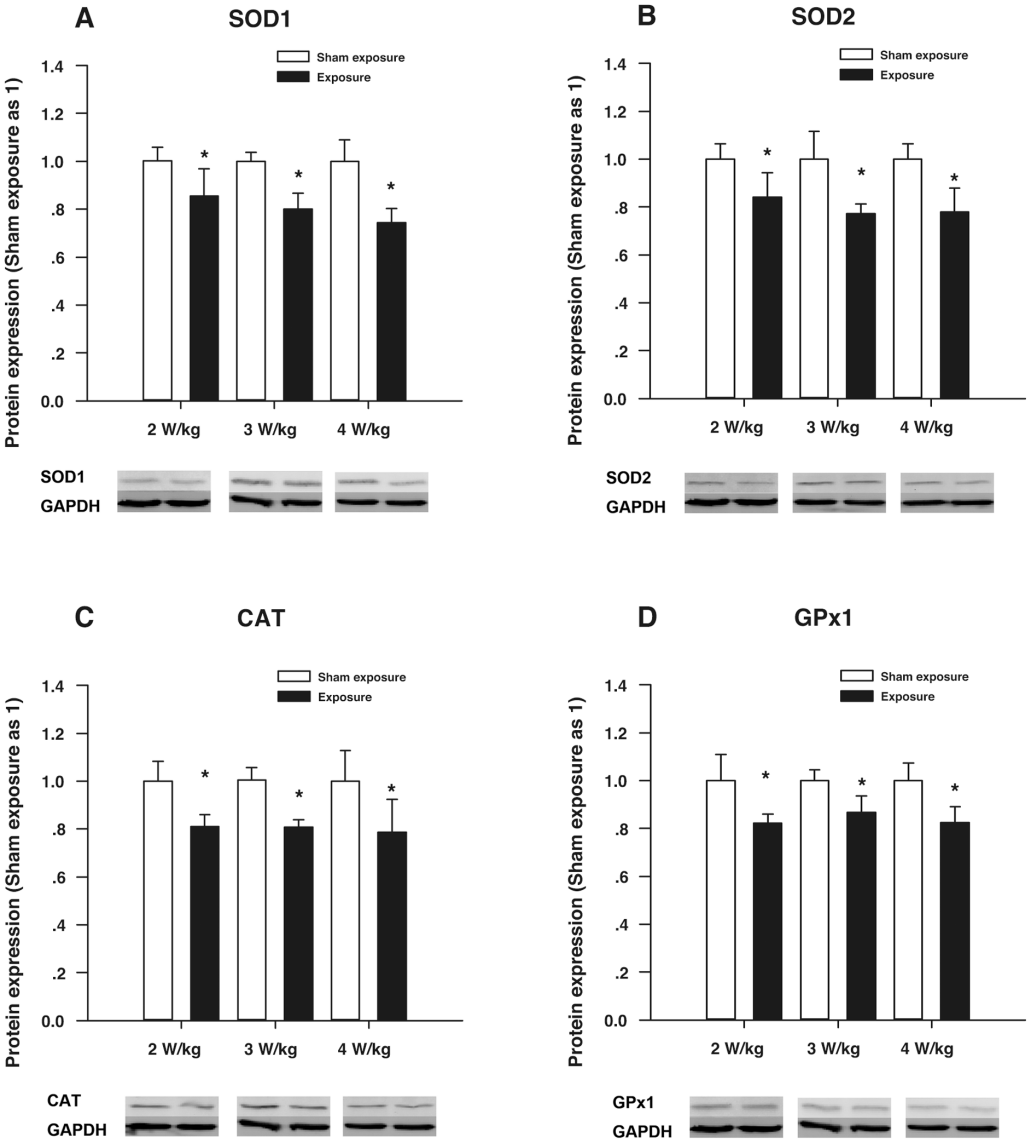
Results of Western blot assay in the HLE B3 cells exposed to RF. (**A**) The expression levels of the SOD1 protein in the HLE B3 cells exposed to RF (SAR: 2, 3, or 4 W/kg) for 1 h. (**B**) The expression levels of the SOD2 protein in the HLE B3 cells exposed to RF (SAR: 2, 3, or 4 W/kg) for 1 h. (**C**) The expression levels of the CAT protein in the HLE B3 cells exposed to RF (SAR: 2, 3, or 4 W/kg) for 1 h. (**D**) The expression levels of the GPX1 protein in the HLE B3 cells exposed to RF (SAR: 2, 3, or 4 W/kg) for 1 h. *P<0.05, as compared with the corresponding sham-exposure subgroups.

Figure 5 demonstrates the relative protein expression levels of the four antioxidant enzymes of the HLE B3 cells exposed to RF for 1 h. In Figure 5A, the SOD1 protein expression levels of the three subgroups exposed to RF (SAR: 2, 3, and 4 W/kg) are significantly reduced compared with the corresponding sham-exposure subgroups (*P*<0.05). In Figure 5B, the SOD2 protein expression levels of the three subgroups exposed to RF (SAR: 2, 3, and 4 W/kg) are significantly lower than those of the three corresponding sham-exposure subgroups (*P*<0.05). In Figure 5C, the CAT protein expression levels of the subgroups exposed to RF (SAR: 2, 3, and 4 W/kg) are obviously decreased compared with the three corresponding sham-exposure subgroups (*P*<0.05). In addition, as shown in Figure 5D, the expression of the GPx1 protein in the three subgroups exposed to RF (SAR: 2, 3, and 4 W/kg) is significantly lower compared with the three corresponding sham-exposure subgroups (*P*<0.05).

## Discussion

### Enhanced Oxidative Stress Occurs in HLE B3 Cells Exposed to Low-intensity 1.8 GHz RF

Oxidative stress can be defined as a state of imbalance between the factors that generate reactive oxygen radicals (e.g., superoxide or hydroxyl radicals) and the factors that protect cellular macromolecules from these reactants, including antioxidants such as SOD, CAT, and GPx. Products of normal cellular physiology generate ROS, as well as various exogenous sources. Low levels of ROS are typical within both the cell and the higher order tissue and organ systems. Some ROS (superoxide and H_2_O_2_ in particular) are required to support natural cellular functioning and to regulate intracellular signaling. Under normal metabolic conditions, the balance between cellular antioxidant defense and ROS generation is maintained, but excessive ROS generation (or reduced ROS regulation) can result in oxidative stress and severely impair the cell and lead to macromolecular damage, dysfunction, and death [34], [35]. Hence, excessive ROS generation is an important biomarker of oxidative stress.

Oxidative stress also causes lipid peroxidation and the formation of reactive aldehydes. The effects of lipid peroxidation include a loss of fluidity, a decrease in electrical resistance, reduced protein mobility in the membrane, and increased phospholipid exchange between the bilayers of the membrane. The breakdown of cell membranes in the worst case causes inactivation of membrane-bound enzymes and events that are catastrophic to the normal function of cells. The aldehydes, including MDA, that are produced as a consequence of lipid peroxidation are potentially harmful. MDA has been shown to cross-link and aggregate membrane proteins. It also leads to oxidation of polyunsaturated fatty acids and thus serves as a reliable biomarker of oxidative stress-mediated lipid peroxidation [4].

Oxidative stress results in macromolecular damage and is implicated in various disease states [34]. Accumulating evidence indicates that ROS directly interacts with critical signaling molecules to initiate signaling in a broad variety of cellular processes, such as proliferation and survival (MAP kinases, PI3 kinase, phosphatase and tensin homolog deleted from chromosome-10, and protein tyrosine phosphatases) [36]. Thus, cellular proliferation and survival can be used as an effective biomarker for oxidative stress [37]–[39].

The present study found that the levels of ROS production and MDA increased and the cellular viability decreased in the HLE B3 cells exposed to 1.8 GHz RF. The results of the three biomarkers (ROS, MDA, and cellular viability) demonstrated that enhanced oxidative stress occurs in HLE B3 cells exposed to 1.8 GHz RF at three different exposure time points and three different exposure intensities. The increased oxidative stress occurred even when the exposure intensity was SAR 2 W/kg, which approaches exposure levels typically associated with the use of cellular phones [3]. Under conditions of oxidative stress, free radicals that are not reduced or removed from the cellular environment can cause damage to all cellular macromolecules, including nucleic acids, lipids, and proteins [35]. The results of the present study may explain some effects reported in previous investigations of our laboratory. For example, Yu *et al*. found that RF exposure could induce expression of heat shock protein 27 (Hsp27) and Hsp70 and the activation of extracellular regulated protein kinases 1/2 and c-Jun N-terminal kinases 1/2 in human LECs, suggesting that nonthermal RF exposure could induce the stress response in human LECs [40]. Yao *et al.* reported that 1.8 GHz RF could induce DNA damage and a decline in cellular proliferation in human LECs [41], [42]. Studies conducted by Li *et al.* and Sun *et al.* showed an increase of Hsp70 and heterogeneous nuclear ribonucleoprotein K expression in human LECs exposed to1.8 GHz RF [43], [44]. Those effects may be related to the increased oxidative stress.

**Increased ROS Production may be Related to Down-regulation of Four Antioxidant Enzyme Genes in HLE B3 Cells Exposed to RF**

ROS molecules include superoxide anions, hydroxyl radicals, and H_2_O_2_. They can be generated endogenously by several enzymatic systems or exogenously from the environment [45]. Mitochondria are an important source of endogenous ROS in the majority of cell types. ROS production contributes to mitochondrial damage in a range of pathological changes and is also important in redox signaling from the organelle to the rest of the cell [46], [47]. Mitochondria are important for integrating many important metabolic activities and signaling pathways in the life and death of a cell. Normal aerobic cells use oxidative phosphorylation to generate ATP, which supplies energy for metabolism. To drive ATP production, electrons are passed along the electron transport chain, with some leaking as superoxide during the process. It is estimated that during normal respiration, intramitochondrial superoxide concentrations can reach 10^−12^ M. This extremely high level of endogenous superoxide production dictates that mitochondria are equipped with antioxidant systems to prevent severe oxidative injury to mitochondria and to maintain normal mitochondrial functions [48]. The antioxidation defense system of cells includes nonenzymatic (e.g.,glutathione, vitamin C, vitamin E, and carotenoids) and enzymatic systems [45].

SODs, CAT, and GPXs are important components of antioxidant enzymes [45]. SODs, the major antioxidant defense systems against O_2_^•–^, consist of three isoforms of SOD in mammals: cytoplasmic Cu/ZnSOD (SOD1), mitochondrial MnSOD (SOD2), and extracellular Cu/ZnSOD (SOD3) [49]. SOD2 is an important antioxidant enzyme that scavenges superoxide anion radicals in mitochondria. It is highly regulated and is encoded in the nucleus by the *SOD2* gene located on chromosome 6q 25. SOD2 is the only enzyme that is essential for the survival of life in the aerobic environment under physiological conditions. This critical function may be due to the strategic location of SOD2 in the mitochondria matrix [48]. SOD1 is the major intracellular SOD and mainly present in the cytosol, with a smaller fraction in the intermembrane space of mitochondria. The human *SOD1* gene is localized on the 21q 22.1 region of chromosome 21 [49]. The function of SODs is to catalyze the conversion of O_2_^•-^ to H_2_O_2_, which may participate in cell signaling. In addition, SODs play a critical role in inhibiting oxidative inactivation of nitric oxide, thereby preventing peroxynitrite formation and endothelial and mitochondrial dysfunction [48], [49]. CAT is a dismutase, which reduces one H_2_O_2_ to H_2_O and oxidizes a second H_2_O_2_ to O_2_, which is exclusively localized in the peroxisomes in mammalian cells. A major role of CAT is likely to remove H_2_O_2_ produced during α-oxidation of fatty acids in peroxisomes [50]. GPX1 is one of the most abundant members of the GPx enzyme family and is present in the cytosol and the mitochondria of all cells. GPX1 is a crucial antioxidant enzyme involved in preventing harmful accumulation of intracellular H_2_O_2_. It has been found to be more effective than catalase in removing intracellular peroxides under many physiological conditions [51].

Several in vivo and in vitro investigations have reported that RF can induce oxidative stress (increased ROS or MDA levels) [2]–[5], [15], [18], [52]. Although measurements of some antioxidant enzymes (SOD, CAT, and GPx enzymes) have revealed decreased activities at the biochemical level [15], [18], [52], the reasons for the enhanced oxidative stress induced by RF exposure at the molecular level have not been elucidated. The present study detected enhanced oxidative stress (increased ROS and MDA levels and reduced cellular viability). Moreover, it showed that the expression levels not only of the *SOD1, SOD 2, CAT, and GPX1* genes, but also those of the SOD1, SOD 2, CAT, and GPX1 proteins were significantly diminished in the HLE B3 cells exposed to 1.8 GHz RF compared with the HLE B3 cells sham-exposed to RF, even when the RF exposure intensity was SAR 2 W/kg. The results suggest that RF may affect the expression of antioxidant enzyme genes (*SOD1, SOD 2, CAT,* and *GPX1* genes). The decrease in the expression levels of the four antioxidant enzyme genes and the proteins might result in an imbalance between oxidants and antioxidants because excessive endogenous ROS from mitochondria cannot be scavenged in time. As a result, ROS production was significantly elevated in the HLE B3 cells exposed to 1.8 GHz RF.
